# Kilometers Long Graphene-Coated Optical Fibers for Fast Thermal Sensing

**DOI:** 10.34133/2021/5612850

**Published:** 2021-03-18

**Authors:** Yiyong Guo, Bing Han, Junting Du, Shanshan Cao, Hua Gao, Ning An, Yiwei Li, Shujie An, Zengling Ran, Yue Lin, Wencai Ren, Yunjiang Rao, Baicheng Yao

**Affiliations:** ^1^Key Laboratory of Optical Fiber Sensing and Communications (Education Ministry of China), University of Electronic Science and Technology of China, Chengdu 611731, China; ^2^Research Centre of Optical Fiber Sensing, Zhejiang Laboratory, Hangzhou 310000, China; ^3^Optical Fiber Co., Ltd., ZTT Group, Nantong 226009, China; ^4^Carbonene Technology Co., Ltd, Deyang 618000, China; ^5^Optical Science and Technology Ltd., China National Petroleum Corporation, Chengdu 610041, China; ^6^Cavendish Laboratory, University of Cambridge, CB3 0HE, UK; ^7^Shenyang National Laboratory for Materials Science, Institute of Metal Research, Chinese Academy of Sciences, Shenyang 110016, China

## Abstract

The combination of optical fiber with graphene has greatly expanded the application regimes of fiber optics, from dynamic optical control and ultrafast pulse generation to high precision sensing. However, limited by fabrication, previous graphene-fiber samples are typically limited in the micrometer to centimeter scale, which cannot take the inherent advantage of optical fibers—long-distance optical transmission. Here, we demonstrate kilometers long graphene-coated optical fiber (GCF) based on industrial graphene nanosheets and coating technique. The GCF shows unusually high thermal diffusivity of 24.99 mm^2^ s^−1^ in the axial direction, measured by a thermal imager directly. This enables rapid thermooptical response both in optical fiber Bragg grating sensors at one point (18-fold faster than conventional fiber) and in long-distance distributed fiber sensing systems based on backward Rayleigh scattering in optical fiber (15-fold faster than conventional fiber). This work realizes the industrial-level graphene-fiber production and provides a novel platform for two-dimensional material-based optical fiber sensing applications.

## 1. Introduction

Winning the Nobel Prize in physics of 2009, optical fibers have been a cornerstone of global information networks [1]. Besides applications in telecommunication, optical fiber-based sensing technology possesses the unique capability to measure the spatial and temporal map of environmental quantities such as temperature, strain, pressure, and electromagnetic fluctuations remotely [2, 3], owing to the kilometers long light transmission. In recent years, distributed fiber optic sensors have spurred wide advances worth billions of dollars, ranging from geophysical exploration and remote security to smart city [4–6]. For meeting the continuously increasing requirement of sensing performance, the combination of advanced materials and optical fibers becomes a significant route.

Graphene with exceptional electric, photonic, mechanic, and thermal properties [7–14] is a broadly attractive material for fiber functionalization [15]. The incorporation of graphene and optical fibers has brought breakthroughs across lasers [16–19] and modulators [20–22] to sensors [23–26]. However, previous graphene-fiber implementations were limited to micrometer-sized samples, until meters long graphene-fiber hybrid production in 2019 [27]. Nevertheless, it is still far from kilometers long graphene fiber for systematic sensing out-of-lab.

Here, we demonstrate a way to produce kilometers long graphene-coated optical fiber (GCF) by spraying graphene nanosheets [28] on optical fiber in the industrial fabrication process. By spraying and molding graphene-acrylate hybrid (30%-wt) on the optical fibers, we produce a graphene coating layer on-fiber with high quality, verified by Raman spectroscopy and electrical measurement. This GCF demonstrates excellent thermal diffusivity *α* ≈ 24.99 mm^2^ s^−1^, which is 30-fold larger than conventional silica fibers (*α* ≈ 0.83 mm^2^ s^−1^). As a result, we experimentally reveal that it shows a unique advantage for fast optical temperature measurements, accelerating the thermooptical response over 18-folds in fiber Bragg grating sensors and over 15-folds in long-distance distributed fiber sensing systems.

## 2. Results

**Figure 1:**
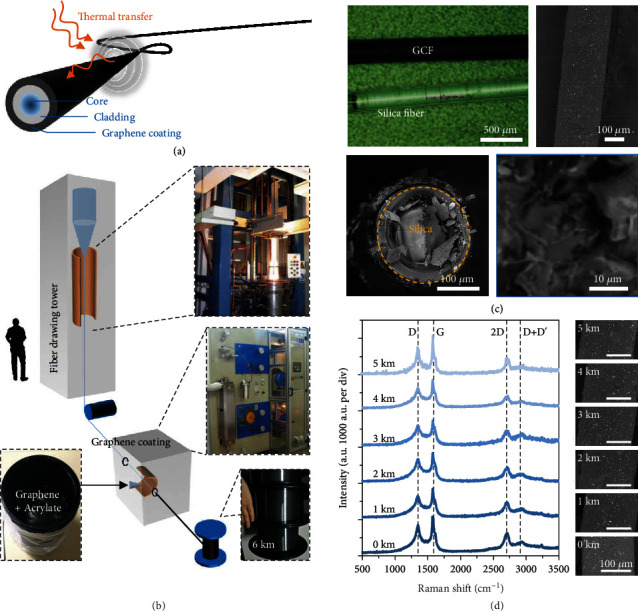
Conceptual design, industrial fabrication, and characterization of the GCF. (a) Schematic diagram of the GCF, 20 *μ*m thick graphene-acrylate hybrid is coated on the silica fiber. It can accelerate heat transfer along the axial direction, enhancing the optical response of the fiber. (b) Industrial fabrication of the kilometers long GCF. Fiber drawing and graphene coating are finished in the assembly line automatically. (c) Microscopic pictures of the GCF; here, scale bars are marked inside. (d) Measured Raman spectra and side-view SEM pictures at different locations along the long GCF, verifying the consistent quality of the graphene and the uniformity of the coating.

Figure 1(a) demonstrates the concept and structure of our long-distance GCF. Conventionally, optical fiber is composed of silica core, silica cladding, and polymer coating, from inside to outside. Thus, the thermal diffusivity of fiber is intrinsically low [29]. Graphene with exotically high thermal conductivity (>2000 Wm^−1^ K^−1^) [30–32] may help to improve the thermal diffusivity of fiber, but to integrate graphene in silica core or cladding will induce considerably high optical loss (several dB per centimeter [27]), and the fabrication meets technical challenges. Thus, we replace the external polymer coating with graphene nanosheets, which can also dramatically enhance heat transfer in the axial direction, via industrially implementable preparation (Figure 1(b)): Kilometers long optical fiber is drawn from silica preform in a drawing tower, with drawing speed 10 m/s; then, the prepared graphene-acrylate hybrid is sprayed directly on the surface of fiber, forming the graphene coating. In the coating material, the content of graphene is 30% in weight. Such optimized graphene content offers enough lubrication, avoids nozzle clogging in production, and ensures good flexibility and strength after solidification. We provide more details about material preparation in Section S1. The coating speed is comparable to the fiber drawing; thus, we can produce 30 km long GCF in 1 hour. Besides, considering that graphene would absorb ultraviolet light, we use high ultraviolet power (3 kW) in the curing process. Thanks to the industrial coating scheme based on jet spray, the cost of the GCF is only 10 dollars per km (2.5 dollars for graphene material and 7.5 dollars for fiber fab). In this figure, we show the picture of 6 km long GCF. Thanks to the industrial scheme, production of the GCF can be easily scaled up to thousand kilometers.

By using optical and scanning electron microscopy, we image the GCF in Figure 1(c). It is clearly observed that the GCF is black in color, due to the graphene coating. The fast coating process also enables the surface of the GCF uniform, and the thickness of the graphene coating layer is ≈20 *μ*m. Here, we also illustrate the sectional view of the GCF and zoom the graphene coating region in. The graphene nanosheets with size of 10~30 *μ*m link and stack with each other randomly. In the cross-sectional SEM picture, the small fragments are induced by the fiber cutting. The dark fragments on the surface are graphene nanosheets, while the bright fragments are silica. The left panel of Figure 1(d) plots the Raman spectra of the graphene on the GCF, measured by using a 514 nm laser, at different spatial locations, from 0 km to 5 km. The position of the G peak is 1576 cm^−1^ on average, with deviation <±1 cm^−1^ and full width at half maximum <30 cm^−1^. The position of 2D peak is 2706.4 cm^−1^ in average, with deviation <±2 cm^−1^ and half-linewidth <70 cm^−1^. Characterization of the G and 2D peaks verifies that the graphene in the coating is in a form of graphene nanoplatelets [33]. Besides, a relatively strong D peak at 1350 cm^−1^ and a relatively high D+D′ peak at 2930 cm^−1^ also indicates that there is the presence of boundaries and defects due to its fragmented nature [34]. Such Raman spectroscopic analysis also suggests that the quality of the graphene keeps well along the long fiber. To further demonstrate the uniformity, we also show the scanning electronic microscopic images at these locations. It verifies that the thickness and carbonation of the coating are uniform.

**Figure 2:**
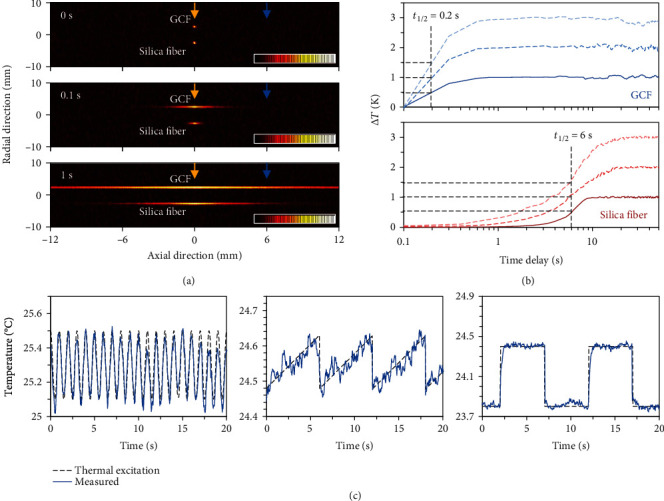
Direct measurement of the thermal response along the GCF. (a) From top to bottom, the thermal images when heating the GCF and the silica fiber 0 s, 0.1 s, and 1 s. Here, the yellow arrow marks the heating point, and the blue arrow marks the point 6 mm away from the heater. Color bar: temperature, 24°C to 25°C. (b) Measured temperature at the point 6 mm away from the heater (see blue arrow in (a)). Upper panel: GCF; bottom panel: commercial silica fiber. Here, the black dashed lines highlight *t*_1/2_. (c) Examples that the GCF measures fast thermal oscillations in varied waveforms. From left to right: sinusoidal, triangle, and square.

To investigate the thermal conduction enhancement, we heat a point on the GCF and on a section of silica fiber simultaneously in a vacuum, and we detect the thermal response by using a thermal imager.  Figure 2(a) maps the images measured at different time delays; here, both the GCF and the silica fiber are fixed in an insulation chamber with controlled macroscopic temperature 24°C, while the heater probe in 0.1 mm size offers stable temperature 25°C (yellow arrow). The comparisons clearly show that the spread of the temperature field along the GCF is faster than the commercial silica fiber: when time delay is 0.1 s, the bright region of GCF (silica fiber) is ≈1.5 mm (≈0.3 mm), and when time delay is 1 s, the bright region of GCF (silica fiber) is >10 mm (≈2 mm). In more detail, we record the temperature dynamics at the point 6 mm away from the heater (blue arrow); Figure 2(b) plots the measured traces. In the measurement, the power of the heater is constant (2 W) for each case, providing constant temperatures. When temperature increases by 1°C, 2°C, and 3°C for transfer distance *L* = 6 mm, the time required for half of the maximum temperature rise *t*_1/2_ of GCF is 0.2 s, while *t*_1/2_ of a silica fiber is 6 s. Referring to the temperature distribution model [35, 36], the thermal diffusivity *α* = 1.38 *L*^2^/*πt*_1/2_, we estimate the diffusivity of the GCF in axial direction *α*_GCF_ = 24.99 ± 1.2 mm^2^/s, 30-fold higher than the silica fiber without polymer coating (*α*_silica fiber_ = 0.83 mm^2^/s). In measurements, systematic error is ±0.01 s and ±10 mK; this thermal diffusivity of the graphene nanosheet composite also meets the predictions in previous studies [37, 38]. Considering the relationship of thermal diffusivity *α* and thermal conductivity *K* = *αρC*_*p*_, we estimate the thermal conductivity of the GCF *K*_GCF_ ≈ 26.3 Wm^−1^ K^−1^ in the axial direction, about 19-fold higher than the silica fiber (1.4 Wm^−1^ K^−1^ [39]); here, *ρ* = 1.5 g/cm^3^ is the density of the graphene coating, and *C*_*p*,graphene_ = 0.7 Jg^−1^ K^−1^ is the thermal capacity of the graphene coating (*C*_*p*,silica_ = 0.68 Jg^−1^ K^−1^ [30]). Such rapid response enables the GCF to percept minor temperature fluctuations in dynamics; we show several examples in Figure 2(c), by adding thermal signals in sinusoidal (1 Hz repetition, 25.1°C to 25.5°C), triangle (1/6 Hz repetition, 24.48°C to 24.62°C), and square (1/10 Hz repetition, 23.8°C to 24.4°C) waveform. The detected temperature at 20 mm away can well follow the real temperature alterations.

**Figure 3:**
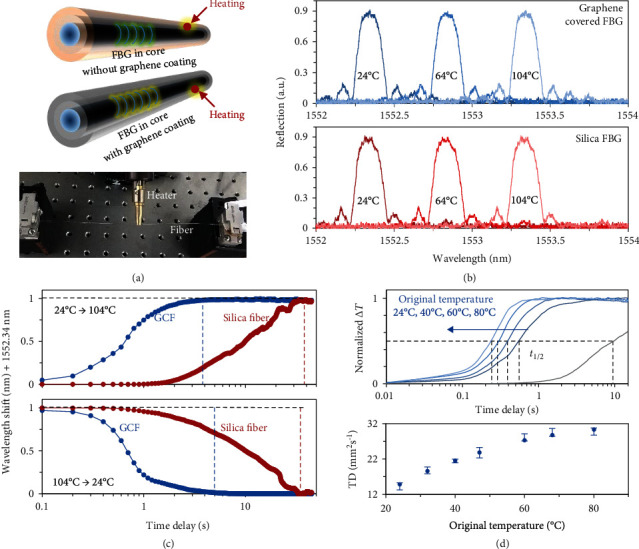
Thermooptical response of the graphene-coated FBG. (a) Schematic scheme of the measurement: same Bragg gratings are written in the core of the GCF and the silica fiber, and a heat probe is used to control the temperature. (b) Central wavelength of the in-core FBGs redshifts when increasing the temperature in fiber. The graphene coating does not influence the sensitivity (12.5 pm/K). (c) Time-dependent wavelength shift of FBGs in the GCF (blue dots) and in the typical silica fiber (red dots), driven by the temperature alteration between 24°C and 104°C. The blue and red dashed line marks that the wavelength shift approaches stability. (d) A higher environmental temperature enables lower delay of thermooptical response. Here, TD means optically measured thermal diffusivity. Error bar denotes the measurement uncertainty.

In fiber sensing applications, the thermooptical response relies on not only the time delay in the axial direction but also the radial direction, from the fiber surface to the core. We simulate the thermal field diffusions in Section S2. For calibrating the practical thermooptical response, we write Bragg gratings in the fiber core of the GCF and use the GCF-based fiber Bragg grating (FBG) in temperature sensing (Figure 3(a)). In this measurement, the temporal sampling rate is >100 Hz, which traces the thermooptical in high resolution. When temperature changes the refractive index *n* or the grating period *Λ* in fiber, the reflected interference peak of a FBG shifts, meeting the relation *λ* = 2*nΛ*; here, *λ* is the Bragg wavelength [40]. In room temperature (24°C), *Λ* = 531.6 nm, *n* = 1.46, *λ* = 1552.34 nm. Then, we heat a point on the GCF, and the thermal transfer distance in the axial direction is 8 mm. Figure 3(b) plots the reflection spectra of the graphene-coated FBG (upper panel) and a typical silica FBG (bottom panel). When heating the fibers from 24°C to 104°C and waiting until stable, the reflected peak of both the graphene-coated FBG and the silica FBG redshifts, from 1552.34 nm to 1553.34 nm. The two types of fiber share the same temperature sensitivity, 12.5 pm/K linearly, because their material and geometry in the fiber core are the same.

As aforementioned, the thermal transfer efficiency of the GCF and the silica fiber is distinguished. We compare the temporal responses of them in Figure 3(c), by using a frequency counter with a sampling rate 1 kHz. When heating the samples from 24°C to 104°C immediately, stable-to-stable response delay of the GCF-based FBG is 4 s for Δ*T* = 80°C, and *t*_1/2_ = 0.6 s. This suggests that the optically tested thermal diffusivity of the GCF is 14.81 mm^2^/s, which is over 18-fold higher than the silica fiber (stable-to-stable delay 40 s, *t*_1/2_ = 10 s, optically measured thermal diffusivity 0.81 mm^2^/s). Moreover, we measure the time delay when the temperature decreases back. From 104°C to 24°C, the GCF spends 5 s, while the silica fiber needs 36 s. Such a fast response is statically verified in repeated measurements, the uncertainty of the delay when temperature tunes up and down on the GCF is less than ±0.2 s. We also note that the thermooptical response speed of the GCF increases with environmental temperature. Figure 3(d) illustrates the measured result: when the environmental temperature ranges from 24°C to 80°C, for the same additional increment +20°C, the optically measured delay *t*_1/2_ decreases from ≈0.6 s to ≈0.3 s (the upper panel). Correspondingly, the optical sensed thermal diffusivity increases from ≈14.81 mm^2^/s to ≈29.61 mm^2^/s.

**Figure 4:**
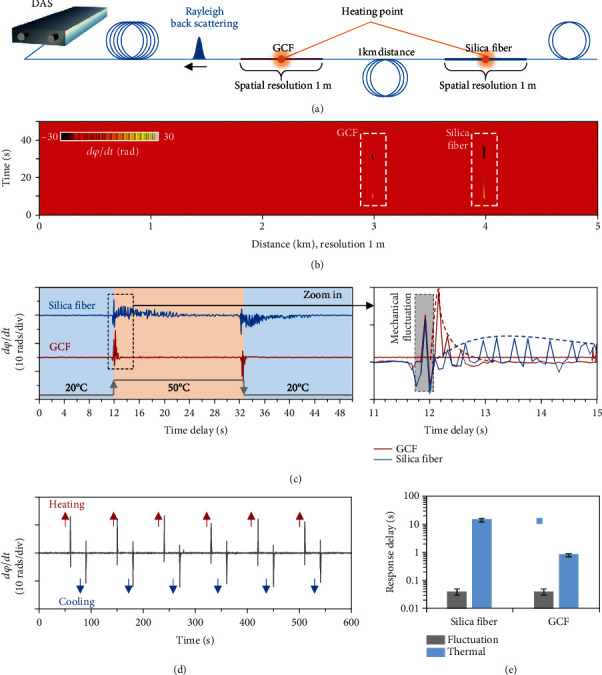
Performance of the GCF in distributed temperature sensing based on *φ*-OTDR. (a) Experimental setup; here, a commercial *φ-*OTDR instrument provides the pump laser and detects the reflected Rayleigh backscattering. A section of GCF is linked in a 5 km long fiber system, and we heat two separate points simultaneously. Spatial resolution of this *φ-*OTDR is 1 m. (b) Measured temperature dynamics of 1 m long GCF (at the 3 km location) and 1 m long silica fiber (at the 4 km location), respectively. (c) Temporal response of the GCF (top panel) and the silica fiber (bottom panel) from 0 s to 50 s. (d) Fast sensing performance of the GCF by repeated measurements. (e) Comparison of the fast sensing performance between the GCF and the silica fiber.

Finally, we use the GCF in both distributed temperature sensing and test its performance. To better measure the fast response of a system, we use a commercial phase-sensitive optical time domain reflectometry (*φ*-OTDR) system. Compared to the distributed temperature systems based on anti-Stokes backward scattering [41], this instrument does not need signal integration temporally, thus enabling a large data sampling rate (25 Hz), which is fast enough to capture the thermal dynamics in fiber. We also discuss the results of different distributed fiber sensing systems in Section S3.  Figure 4(a) illustrates the experimental setup. A commercial *φ*-OTDR instrument (Optical Science and Technology Ltd., uDAS) is employed to compare the performances of the GCF with the silica fiber under the same condition (core/cladding diameter 9/125 *μ*m). The two fiber sections are spliced in a 5 km long fiber system, at the location of 3 km away from the instrument. In order to separate the responses of the GCF and the silica fiber, an additional 1 km long silica fiber is used to link the two sections. In the measurement, we adopt a 1 m spatial resolution to minimize the pulse uncertainty and use a 12.5 Hz sampling rate for obtaining enough signal-to-noise ratio (SNR). In the sensing process, the *φ*-OTDR detects the phase difference change of the reflected Rayleigh backscattering in fiber, which relies on the temperature alteration.

We first heat the GCF and the silica fiber simultaneously from 20°C to 50°C. A moment later, we cool their temperature from 50°C down to 20°C back. We map the thermooptical response in Figure 4(b). In the heating process, the detected phase difference change *dφ*/*dt* > 0; oppositely in the cooling process, the detected phase difference change *dφ*/*dt* < 0. When the temperature is stable, *dφ*/*dt* = 0. Hence, we can conveniently obtain the response delay from one thermal state to another thermal state. It is obvious that the GCF section responds much faster than the silica fiber section.  Figure 4(c) shows their temporal traces more in detail. Once putting the heater on fiber, we observe a sharp oscillation immediately. This is a mechanical fluctuation signal induced by the fiber heater. Its temporal duration is <0.02 s. Meanwhile, the fibers begin to feel the heat. In the GCF section, benefiting from its fast thermal conduction, the temperature of the whole section changes rapidly from 20°C to 50°C (stable to stable); the optical measured delay of the GCF section is ≈0.8 s. In comparison, the silica fiber responds slowly (temporal delay ≈ 12 s). Such fast thermal response of the GCF is repeatedly measured as shown in Figure 4(d). We also note that the graphene on-fiber coating does not deteriorate the sensitivity, because the graphene does not influence the light propagating in the fiber core. By periodically heating and cooling the GCF, we plot its phase difference response in Figure 4(c). In this measurement, data error is <5%.  Figure 4(e) summarizes the fast sensing performance. For the fast distributed sensing system for temperature tracing, the graphene-coated fiber can respond 15 times faster than the silica fiber. We also note that the spatial resolution of the sensing system is determined by the signal modulation; hence, using the GCF would not improve its positioning accuracy. Besides the *φ*-OTDR, we also demonstrate that our GCF responds faster than silica fiber in the distributed temperature sensing (DTS) system based on Raman backscattering (see extended data in Section S3).

## 3. Discussion

In a nutshell, we report the kilometers long GCF, which could be mass-produced, via coating graphene nanosheets on the surface of silica fibers industrially. Such GCF demonstrates fast thermal response, with thermal diffusivity ≈ 24.99 mm^2^/s in the axial direction, 30-fold higher than conventional silica fiber. Leveraging this new fiber can enable detecting acceleration over 18-fold improvement for the FBG temperature sensor at one point, or 15-fold improvement for long-distance distributed fiber sensing system. Such fast response also demonstrates high reliability and robustness in repeated measurements. This work builds a bridge for the industrial combination of graphene and fiber optics, which offers not only inspiration for the combination of other 2D materials and optical structures but also a novel platform for high-performance optic-fiber temperature sensing, which may bring unpredicted evolution for applications such as deep-earth/deep-sea exploration, remote safety monitoring, and fire warning.

## 4. Materials and Methods

### 4.1. Fabrication and Characterization of the GCF

First, we mixed and stirred the graphene dispersion and the acrylate (graphene 30 wt%). This hybrid is sprayable by using an industrial fiber coater, under 120 kPa pressure. Optical fibers were fabricated directly in the drawing tower. Then, we controlled the spraying speed to ensure the coating thickness 20 *μ*m. After surface coating, the graphene-acrylate film was exposed to UV light for full solidification. This implementation replaced the conventional polymer coating of a fiber to graphene-based material. We characterized the quality of the graphene coating by using microscopy, electric method, and Raman spectroscopy. The graphene nanosheets on-fiber are distributed tightly, enabling a typical electrical resistance 6 k*Ω*/mm.

### 4.2. Experimental Measurements

(1) Direct measurement of thermal diffusivity of the GCF: A temperature-controlled probe (TEC) was applied to heat a point on the fiber. The heated short fiber sections were pictured by using a lens calibrated thermoimager (Flir 655c), with temperature resolution 0.1 K, and spatial resolution 0.1 mm. (2) Optical measurement by using FBGs: We prepared FBG by using UV writing technique, and then, we coated the graphene-acrylate on the FBG. The FBG was measured by using an optoelectronic demodulator (FAZ Technology I4G), with a sampling rate 1 kHz and frequency resolution 0.1 pm. (3) Measurement based on the *φ*-OTDR: The temperature distribution along the GCF/silica fiber was measured through employing a *φ*-OTDR (Optical Science and Technology Ltd., uDAS), with spatial resolution 1 m and maximum sampling rate 250 Hz.

## Supplementary Materials

Supplementary Materials Section S1: material preparation and characterization. Section S2: experimental setups and extended discussions for FBG testing. Section S3: experimental setup and extended discussions for distributed fiber temperature testing. Figure S1: preparation of the graphene nanosheets in industry. Figure S2: electrical property of the GCF. Figure S3: setups. Figure S4: simulated temperature field diffusions. Figure S5: measured spectral shift trace of the graphene-coated FBG. Figure S6: schematic diagrams of the distributed fiber sensing systems. Figure S7: performance of the GCF for temperature sensing in Raman scattering-based DTS. Table S1: properties of the graphene nanosheets. Table S2: comparison of different GCF fabrication schemes. Table S3: sensing performance comparison of the graphene-coated and gold-coated fibers. References (28, 42–46).
