# Supplementary material for: Kilometers Long Graphene-Coated Optical Fibers for Fast Thermal Sensing
Source: Research (Wash D C). 2021 Mar 18;2021:5612850. doi: 10.34133/2021/5612850 (PMC8000361; doi:10.34133/2021/5612850)
Supplement: Supplementary Materials — Section S1: material preparation and characterization. Section S2: experimental setups and extended discussions for FBG testing. Section S3: experimental setup and extended discussions for distributed fiber temperature testing. Figure S1: preparation of the graphene nanosheets in industry. Figure S2: electrical property of the GCF. Figure S3: setups. Figure S4: simulated temperature field diffusions. Figure S5: measured spectral shift trace of the graphene-coated FBG. Figure S6: schematic diagrams of the distributed fiber sensing systems. Figure S7: performance of the GCF for temperature sensing in Raman scattering-based DTS. Table S1: properties of the graphene nanosheets. Table S2: comparison of different GCF fabrication schemes. Table S3: sensing performance comparison of the graphene-coated and gold-coated fibers. References (28, 42–46). [file 5612850.f1.docx]

**Kilometers long graphene coated optical fibers for fast thermal sensing**

Yiyong Guo^1†^, Bing Han^1,2†^, Junting Du^1†^, Shanshan Cao^3^, Hua Gao^4^, Ning An^1^, Yiwei Li^1,2^, Shujie An^1,5^, Zengling Ran^1,5^, Yue Lin^6,^*, Wencai Ren^7,^*, Yunjiang Rao^1,2,^*, and Baicheng Yao^1,^*

^1^Key Laboratory of Optical Fiber Sensing and Communications (Education Ministry of China), University of Electronic Science and Technology of China, Chengdu 611731, China.

^2^Research Centre of Optical Fiber Sensing, Zhejiang Laboratory, Hangzhou 310000, China.

^3^Optical Fiber Co., Ltd., ZTT Group, Nantong 226009, China.

^4^Carbonene Technology Co., Ltd, Deyang 618000, China.

^5^Optical Science and Technology Ltd., China National Petroleum Corporation, Chengdu 610041, China.

^6^Cavendish Laboratory, University of Cambridge, CB3 0HE, United Kingdom.

^7^Shenyang National Laboratory for Materials Science, Institute of Metal Research, Chinese Academy of Sciences, Shenyang 110016, P. R. China.

^†^These authors contributed equally to this work.

^*^Corresponding authors: [yaobaicheng@uestc.edu.cn](mailto:yaobaicheng@uestc.edu.cn); [yjrao@uestc.edu.cn](mailto:yjrao@uestc.edu.cn); [wcren@imr.ac.cn](mailto:wcren@imr.ac.cn); [yl589@cam.ac.uk](mailto:yl589@cam.ac.uk)

**Section S1.** **Material preparation and characterization**

The graphene nano-sheets are mass-fabricated by using the “Intercalation–exfoliation” technique (*28*). Preparing high quality graphene nano-sheets from graphite needs to weaken the interlayer Van der Waal’s force and avoid the destruction of graphene 2D structure. **Fig. S1A** shows the scheme. First, we mix and heat the intercalant molecules (ICl or IBr) and graphite, to expand the interlayer distance of the graphite. These intercalant molecules could be released afterwards, without breaking the structure of graphene two-dimensionally. Then we anneal the graphite intercalation compound in liquid (ethanol, H_2_O_2_), obtaining graphene nano-sheets. This fabricated process doesn’t need redox, thus keeps the intrinsic structure of pristine graphene, ensuring its high electrical conduction, thermal conduction and good mechanical properties. **Fig. S1B** demonstrates the mass fabrication of graphene nano-sheets by Carbonene Technology. **Fig. S1C** shows scanning electron microscopic images of the graphene nano-sheets, and we conclude the properties of the graphene nano-sheets in the **Table. S1**.

**Fig. S1D** compares the Raman spectra of the GCF and a CVD-graphene deposited fiber. By using a 514 nm laser, we can characterize the quality of graphene via Raman spectroscopy. Here we majorly consider the information from the D peak, the G peak, the 2D peak, the D+D’ peak and the 2D’ peak. The D peak is determined by the iTO phonon scattering at *K* point, demonstrating the defect or boundary density of graphene. In the CVD monolayer graphene sample, the D peak is very weak, suggesting it is defectless. However, due to the graphene nano-sheets nature, the graphene-acrylate hybrid on fiber has a stronger D peak. The G peak is a feature of sp^2^ bonding of carbon atoms, which helps to justify the graphene material. Both the CVD graphene and the graphene-acrylate have an obvious G peak, verifying that they are both in 2D structure. We note that the D peak is not symmetric due to the D’ peak, which is formed by the in-valley scattering majorly (iLO phonon). This is also an evidence that this hybrid material has many defects or boundaries inside. The 2D peak (or G’ peak), located around 2700 cm^-1^, its existence strongly supports that the material is graphene. The location and linewidth of this peak relies on the doping of graphene. Here, the 2D peak of the graphene-acrylate hybrid is relatively weak with location a bit > 2700 cm^-1^, showing that the graphene nano-sheets may contain multilayers. Moreover, in the graphene-acrylate hybrid, there is an observable D+D’ peak, at 2930 cm^-1^, formed by dual-phonon interaction. The intensity of this D+D’ peak is not very high, illustrating the connection of the graphene nano-sheets is tight. Moreover, in Fig. S1E, we show the SEM pictures of GCFs with varied coating thickness. By changing the technical parameter in production, we can easily control the coating thickness. In this work, we mainly produce GCF with graphene-acrylate coating thickness about 20 μm. When the coating is too thin (< 10 μm), the GCF is fragile and easy to break, when the coating is too thick (> 50 μm), the uniformity of the coating is hard to control, and the thermal diffusion of the GCF would deteriorate.

**Fig. S2** shows the measurement of the electrical conductivity of the GCF. First, we build a setup (**Fig. S2A**), by using two electrodes to inject current and estimate the electric resistance (electrode distance 1 cm), and using a lens focused thermal imager (spatial resolution 0.1 mm) to detect the temperature dynamics. **Fig. S2B** plots the measured voltage-current (*U-I*) curve and corresponding resistance of the GCF. The graphene is originally p-doped, then the injected electrons can shift its doping, hence drive its Fermi level of the graphene moving across the Dirac point (at *U* = 18 V). During this process, resistance of the graphene *R_G_* increases first (up to 8 kΩ/mm), and then decrease back (about 6 kΩ/cm), this phenomenon meets previous studies (*42*, *43*). Besides, a 20 V driven voltage can heat 1 mm long GCF from 24 ^o^C to 28 ^o^C (**Fig. S2C**), determined by the *Ohm’s* heating law *E* = *IU*. In production, we tried different graphene-polymer hybrids for optimizing the coating. Specifically, when the proportion (or concentration) of graphene nano-sheets is too high (> 35 wt%), the graphene-polymer would block the nozzle of the coating machine. Oppositely, when the proportion (or concentration) of graphene nano-sheets is too low (< 20 wt%), the thermal response enhancement is not obvious. Besides, we also compare the performances by using different organic binder in **Table S2**.

**Section S2.** **Experimental setups and extended discussions for FBG testing**

**Fig. S3A** shows the experimental setups fabricating the GCF and the graphene coated FBG. Kilometers long GCF is fabricated in the optical fiber drawing assembly line, the temperature of the flame is > 1750 ^o^C. Silica fiber is drawn from standard preform (typically diameter 100 mm), which determines the fabricated fiber is single mode fiber or multimode fiber. Afterwards, we coat the graphene-acrylate film on the surface of the silica fiber. Spraying speed 7.2 mL/s. Then the coated graphene-acrylate film is solidified via UV irradiation (345 nm). On the other hand, we fabricate graphene coated FBGs. A 248 nm ultrafast laser writes periodic gratings in the core of a silica fiber, via using a lithography mask. Such FBGs could be written online, forming FBG string in one dimension, along the fiber. Finally, we can coat graphene-acrylate on the surface of the FBGs, by using the same spraying technique. **Fig. S3B** illustrates the setup to measure thermal conduction on the surface of the GCF. A TEC (Thorlabs TC-200) controlled heating probe (0.1 mm in size) is used to tune one point of the GCF. Then a thermal imager (Flir 655c) is used to capture the map of the temperature field along the fiber. To avoid other thermal conductions or heat radiation in air, the GCF is suspended in vacuum (10-5 torr). By analyzing the time-temperature correlation at a location with distance L away, we can estimate the thermal diffusivity of the GCF. **Fig. S3C** demonstrates the experimental setup to measure the optical response of a graphene coated FBG. A circulator is used to measure the reflected spectrum. A tunable laser scans the wavelength region 1550 nm to 1560 nm, the data is synchronized in a FBG demodulator, which contains a frequency counter (sampling rate 1 kHz, frequency resolution 0.1 pm).

By using finite-element-method in commercial software *COMSOL*, we show the dynamic temperature fields of a fiber section, with varied thermal conductions of the coating, in **Fig. S4**. First we show the model in geometry, here we assume a 360 K temperature at one point on the top surface of the fiber, and the original temperature of the whole fiber is 300 K. The diameter of the silica part is 125 μm and the graphene coating has a thickness of 20 μm. The thermal conductivity of the silica is 1.4 Wm^-1^K^-1^, density 2.5 g/cm^3^, and heat capacity 920 Jkg^-1^K^-1^, and we type the density of the graphene-coating film 1 g/cm^3^, and heat capacity 1500 Jkg^-1^K^-1^. In this simulation, we demonstrate the temperature distributions at delay of 0.06 s and 0.6 s. We also vary the thermal conductivity of the coating film from 10 Wm^-1^K^-1^ to 1000 Wm^-1^K^-1^, and show the temperature field distribution trends. It demonstrates that a high surface thermal conductivity enables temperature field diffusion on the surface in the axial direction faster than in the radial direction.

**Fig. S5** plots the optical response of the graphene coated FBG in repeated measurements, via heating/cooling it in the temperature region 24 ^o^C ~ 104 ^o^C periodically. Here the heater is placed 8 mm away from the FBG section, the same as the case in the maintext. This figure verifies that the optically measured thermal delay *t_1/2_* in the graphene coated FBG in both heating and cooling process is ≈ 0.6 s, with statistic mean square error < ± 0.1 s.

**Section S3.** **Experimental setup and extended discussions for distributed fiber temperature testing.**

To compare the performances of the GCF and the silica fiber in a long-distance distributed fiber sensing system, we employ a phase-demodulated *φ*-OTDR instrument (Optical Science and Technology Ltd., uDAS). The schematic diagram of the phase-demodulated *φ*-OTDR system is shown in **Fig. S6A**. The ultra-narrow linewidth laser injected light pulses into the sensing fiber with length of *L*, then the phase difference *φ* of the light pulses is

. (S1)

Here, *β* is the propagation constant. When external temperature environment change, the thermal expansion, thermo-optic effect and Poisson effect will cause the fiber length, refractive index and diameter change, respectively. Then, the induced phase difference change of the light pulses under temperature variation is

. (S2)

Here, *n* and *D* are diameter and refractive index of the fiber core, respectively. Hence, with the help of 3×3 phase demodulation (*44*), the external temperature vibration can be detected quantitively by demodulating the phase difference change Δ*φ*.

Distributed temperature sensor (DTS) utilizes spontaneous Raman backscattering in the fiber to detect the environment temperature, which can carry out several kilometers of measurement with high spatial resolution (*45*). Our DTS system is shown in **Fig. S6B** schematically. The 1550 nm signal light from a laser diode (LD) is modulated into pulse light and injected into the sensing fiber through an erbium-doped fiber amplifier (EDFA) and a circulator. As the signal pulse light transmitting in the fiber, the spontaneous Raman scattering, which contains Stokes light and anti-Stokes scattering light, occurs due to the non-uniformity of the material and the thermal vibration effect. Then, the Stokes and anti-Stokes backscattering light return to the circulator and are separated by the wavelength division multiplexer (WDM). The received Stokes backscattering light and anti-Stokes backscattering light are injected into photodetectors, respectively. The photon number of the received Stokes backscattering light and anti-Stokes backscattering light at the position z along the fiber is

$N_{i}=K_{i}\eta\nu_{i}^{4}N_{e}\exp[-(\alpha_{o}+\alpha_{i})L(z)]R_{i}(T), i=S,a$. (S3)

Here, *K_S_* and *K_a_* are the coefficients related to the scattering cross-section of the Stokes and anti-Stokes backscattering light, respectively; *ƞ* is the backscattering factor in the fiber. *ν_S_* and *ν_a_* are the frequency of the Stokes and anti-Stokes backscattering light, respectively; α*_0_*, α*_S_* and α*_a_* are the transmission loss of signal pulse light, Stokes backscattering light and anti-Stokes backscattering light, respectively and *L(z)* is the distance from the signal pulse light input end to the position *z*, which can be determined according to the time delay based on the method of optical time domain reflectometry (OTDR). Moreover, *R_S_(T)* and *R_a_(T)* are the coefficients related to the number of configurations in low and high energy levels of fiber molecules, which can be written as

$\begin{matrix} R_{S}(T)=[1-\exp(\frac{-h\cdot\Delta\nu}{kT})]^{-1} \\ R_{a}(T)=[\exp(\frac{-h\cdot\Delta\nu}{kT})-1]^{-1} \end{matrix}$. (S4)

Here, *h* is the Planck constant, *k* is the Boltzmann constant, *Δν* is the Raman frequency shift in the silica fiber. It can be seen from equation (1) and (2) that the intensity of the anti-Stokes backscattering light is relative to temperature, while the intensity of Stokes backscattering light is relatively insensitive to temperature (**Fig. S6C** and **S6D**). Thus, the environmental temperature can be measured accurately by the ratio of the photon number of anti-Stokes backscattering light to that of the referenced Stokes backscattering light as (*46*)

. (S5)

The intensity of the Raman backscattering light in the fiber is extremely weak, which is 30 dB lower than Rayleigh backscattering light. In measurement, once the stimulated Raman scattering (SRS) excited, it causes the energy transfer from the signal pulse light to Stokes light leading to the impossibility of the accurate long-distance distributed temperature sensing. Meanwhile, due to the received Raman backscattering light intensity in DTS is too weak, the cumulative average method is always adopted to increase the signal-to-noise ratio (SNR) resulting in long measuring time.

**Fig. S7A** illustrates the experimental setup of a DTS. A commercial DTS instrument (AP sensing, N4416A) is employed to compare the performances of the GCF with the silica fiber under the same condition (core/cladding diameter 9/125 μm). The two fiber sections are spliced in a 6 km long fiber system, at the location of 5 km away from the DTS. In order to separate the responses of the GCF and the silica fiber, an additional 200 m long silica fiber is used to link the two sections. In the measurement, we adopt a 1 m spatial resolution to minimize the pulse uncertainty, and use a 30s integral interval window for obtaining enough signal-to-noise ratio (SNR). In sensing, the DTS detects the reflected anti-Stokes light intensity based on spontaneous Raman scattering, which relies on the temperature.

When heating the GCF and the silica fiber simultaneously from 24 ^o^C to 84 ^o^C, we map the thermo-optical response in the DTS, as shown in **Fig. S7B**. By increasing the temperature up (*ΔT* = 60 ^o^C) from room temperature, both the anti-Stokes reflection boost, but the GCF offers an obviously faster response (*t_1/2_* = 60 s) than the silica fiber (delay 300 s). Furthermore, we consider the influence induced by the temporal integral of the signal (in DTS, the temporal integral helps to increase this), which is a tunable parameter in the DTS (**Fig. S7C**).

In this implementation, we heat a point at the fibers, and then keep the temperature 150 s, finally decrease the temperature back. In repeated measurements, we control the temporal integral interval from 0s to 120 s. When the integral interval is 0 s, it reveals the intrinsic thermal response of the GCF and the silica fiber. In this case, time delay *t_1/2,GCF_* ≈ 30 s, and *t_1/2_*,*_silica fiber_* ≈ 280 s, suggesting that the thermal response of the GCF is over 9 times faster than the silica fiber. This number is smaller than the result in FBG testing, because the DTS records the temperature distribution in the whole fiber section rather than one point. When the integral interval is 120 s, the response delay of the GCF *t_1/2_*,*_GCF_* ≈ 112 s, while the response delay of the silica fiber *t_1/2_*, *_silica fiber_* ≈ 350 s. The temporal integral deteriorates the advantage of the GCF relatively, but increases the SNR. **Fig. S7D** discusses the sensing performance. In our DTS, the original SNR without integral average is only 10 dB, but 1 min integral average can suppress the white noise and boost the SNR to 26 dB, with sampling rate 1 Hz. In long distance distributed sensing applications, one needs to balance the performance of time delay and SNR, or develop new fiber structures and materials for further enhancing the intensity of the anti-Stokes scattering of the fiber.

Finally we compare the temperature sensing performance of the GCF with the gold-coated fiber, as it is known that metals especially gold show good thermal diffusion (127 mm^2^/s). Here 200 nm thick gold was sputtered on the surface of the commercial silica fiber (with 20 μm thick polymer productor). Table S3 discusses the measured results. Although the gold coated fiber demonstrates comparable thermal conduction, but its cost is much higher than the GCF.

***Supplementary Figures and Tables***


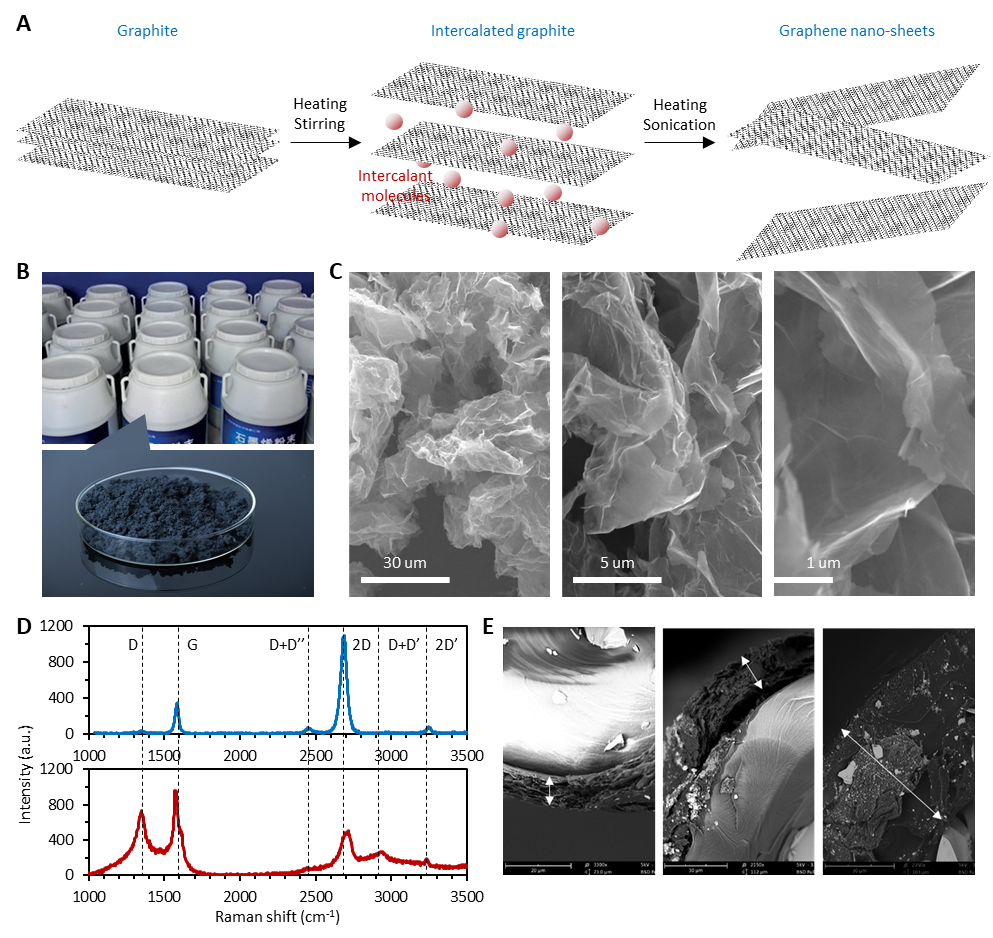


**Fig. S1****. Preparation of the graphene nano-sheets in industry.** **(A)** Fabrication scheme: Intercalation–exfoliation. **(B)** Industrial product of the graphene nano-sheets. **(C)** Scanning electron microscopic images. **(D)** Raman spectra: upper panel, CVD monolayer graphene deposited on fiber; bottom panel: graphene-acrylate hybrid coated on fiber. **(E)** Fabricated GCF with varied coating thickness.

**Fig. S****2.** **Electrical property of the GCF. (A)** Experimental setup for measuring the electrical conduction and electro-thermal response. **(B)** Measured current (blue dots) and resistance (red dots) of the GCF, varying with the driven voltage. Here the results are plotted in repeated measurements. **(C)** The electric current can heat the GCF locally, a higher voltage enables higher local temperature.

**Fig. S****3. Setups. (A)** Schematic diagram of the setup fabricating the GCF and the graphene coated FBG. **(B)** Setup for measuring the thermal response of the GCF directly. **(C)** Setup for measuring the temperature based on optical FBG.


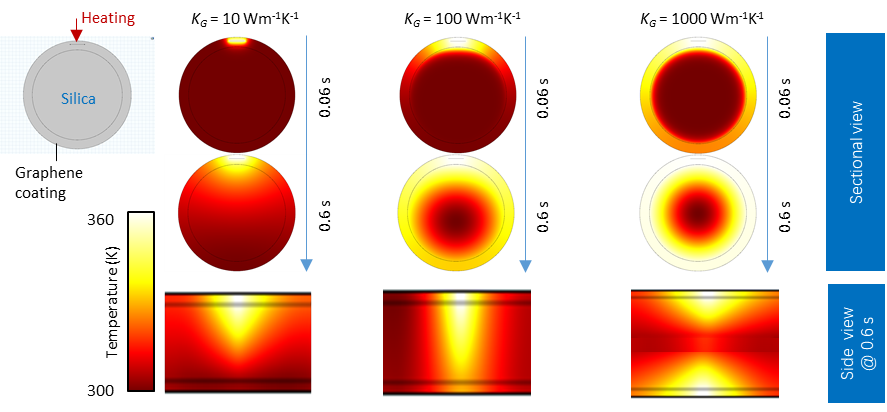


**Fig. S****4. Simulated temperature field diffusions.** Sectional views: From top to bottom, thermal diffusion at 0.06 s and 0.6 s. Side views show the cases at 0.6 s.

**Fig. S****5.** **Measured spectral shift trace of the graphene coated FBG.** In this process, we switch the at-point temperature between 24 ^o^C and 104 ^o^C repeatedly.


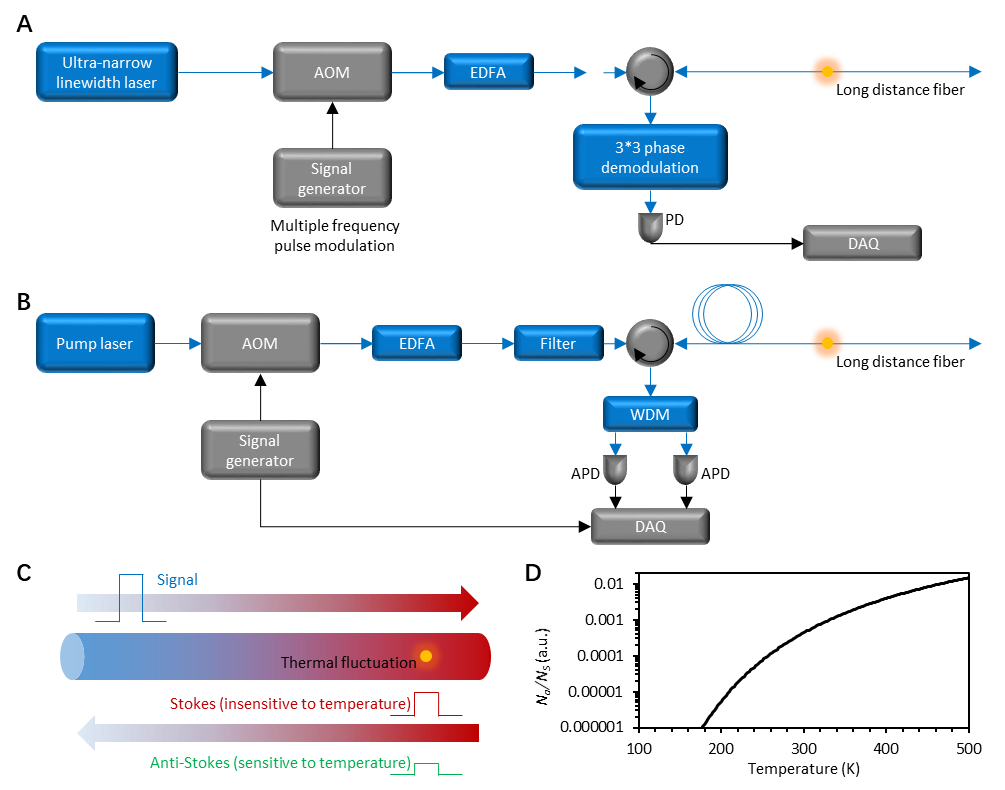


**Fig. S****6. Schematic diagrams of the distributed fiber sensing systems. (A)** φ-OTDR. Spatial sensitivity 1 m, temperature sensitivity 1 ^o^C. **(B)** schematic diagram of a DTS. AOM: acoustic optical modulator, EDFA: erbium-doped fiber amplifier, WDM: wavelength division multiplexer, APD: amplified photodetector, DAQ: data Acquisition. **(C)** Principle of the DTS, the thermal fluctuation only affects the optical intensity of the anti-Stokes backward scattering. **(D)** Calculated result in a typical fiber, a higher environmental temperature enables higher anti-Stokes power.


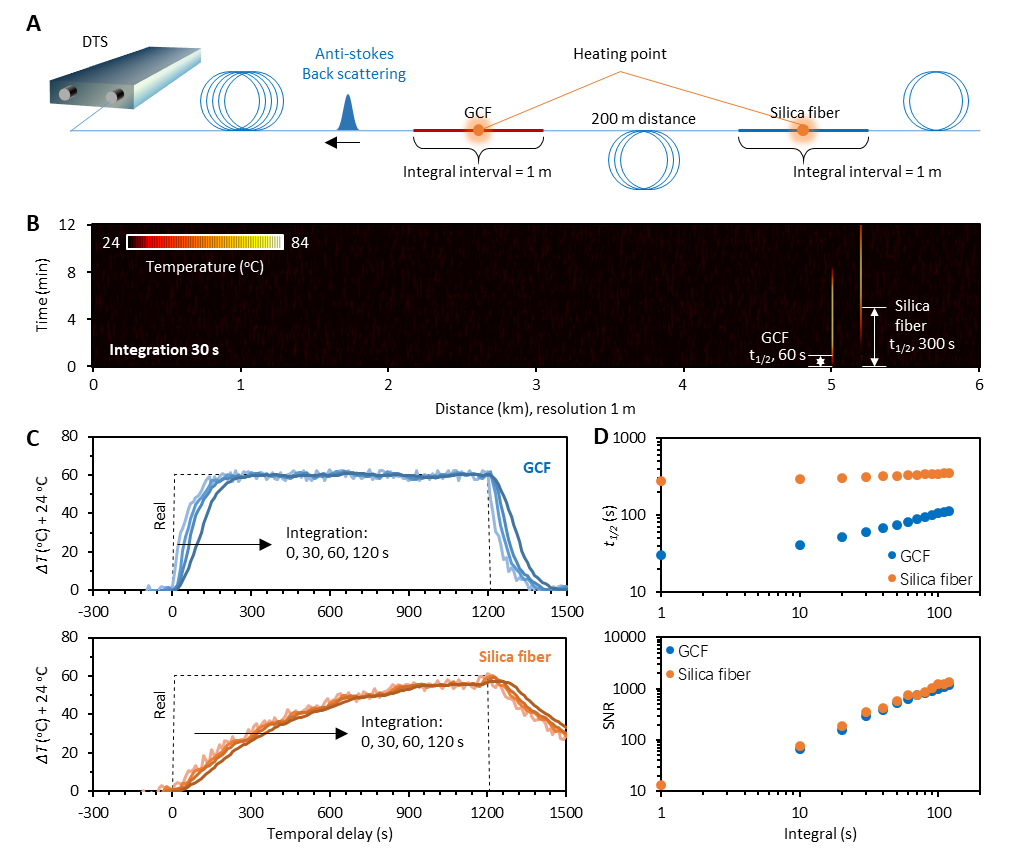


**Fig. S7. Performance of the GCF for temperature sensing in Raman scattering based DTS. (A)** Experimental setup, here a commercial DTS instrument provides the pump laser and detect the reflected anti-Stokes scattering. A section of GCF is linked in a 6 km long fiber system, and we heat two separate points simultaneously. Spatial resolution of this DTS is 1 m. **(B)** Measured temperature dynamics of 1 m long GCF (at the 5th km location) and 1m long silica fiber (at the 5.2 km location), respectively. **(C)** Temporal response of the GCF (top panel) and the silica fiber (bottom panel), when we integrate the signals in a window from 0 s to 60 s. **(D)** Discussion. Top panel: total time delay increases with integral interval. The GCF demonstrates obvious advantage when the integral delay is smaller. Bottom panel: correlation of the integral time and the SNR, which becomes higher with longer integral.

**Table S1. Properties of the graphene nano-sheets**

| **Parameter** | **Typical value** | **Test method** |
| --- | --- | --- |
| **Average Layer** | 1~6 | TEM statistics |
| **Maximum thickness (nm)** | < 3 | TEM statistics |
| **Lamellar size (μm)** | 15 | SEM statistics |
| **Specific surface area (m^2^/g)** | 40~60 | BET nitrogen adsorption |
| **Ash (%)** | < 3 | Burning, 1100 ^o^C |
| **Electrical resistance (S/cm)** | > 700 | Probing test |
| **C-O ratio (wt%)** | >60 | Organic element analysis |

**Table S2. Comparison of different GCF fabrication schemes**

| **Formula** | **Coating&**  **solidification feasibility** | **Minimum**  **bending radius** | **Axial**  **tensile Strength** | **Other notes** |
| --- | --- | --- | --- | --- |
| **Commercial fiber (SMF28e)** | / | < 0.5 cm | 220 kpsi |  |
| **30% graphene**  **+ 70% acrylate** | Yes | ≈ 0.8 cm | 170 kpsi | / |
| **40% graphene**  **+ 60% acrylate** | No  (block nozzle) | / | / | / |
| **30% graphene**  **+ 70% PEGDA** | Yes | ≈ 1 cm | 65 kpsi | Relatively expensive |
| **30% graphene + 70% epoxy acrylate** | No  (hard  to solidify) | / | / | Too soft |
| **30% graphene**  **+ 70% polyimide** | Yes | < 1 cm | 270 kpsi | Alternative for high temperature applications |

**Table S3. Sensing performance comparison of the graphene coated and gold coated fibers**

| **Performance** | **Graphene coated fiber** | **Gold coated fiber** |
| --- | --- | --- |
| ***t_1/2_* in thermal imaging** | 0.2 s | 0.3 s |
| ***t_1/2_* in FBG testing** | 0.6 s | 0.7 s |
| ***t_1/2_* in *φ*-OTDR testing** | 0.8 s | 1.1 s |
| **cost** | 10 dollars/km | 500 dollars/km |

**References and Notes**

44. A. Masoudi, M. Belal, T. P. Newson, A distributed optical fibre dynamic strain sensor based on phase-OTDR. *Meas. Sci. Technol.* **24**, 085204 (2013).

45. R. Sutherland, J. Townend, V. Toy, P. Upton, J. Coussens, M. Allen, L. M. Baratin, N. Barth, L. Becroft, C. Boese, A. Boles, C. Boulton, N. G. R. Broderick, L. Janku-Capova, B. M. Carpenter, B. Célérier, C. Chamberlain, A. Cooper, A. Coutts, S. Cox, L. Craw, M. L. Doan, J. Eccles, D. Faulkner, J. Grieve, J. Grochowski, A. Gulley, A. Hartog, J. Howarth, K. Jacobs, T. Jeppson, N. Kato, S. Keys, M. Kirilova, Y. Kometani, R. Langridge, W. Lin, T. Little, A. Lukacs, D. Mallyon, E. Mariani, C. Massiot, L. Mathewson, B. Melosh, C. Menzies, J. Moore, L. Morales, C. Morgan, H. Mori, A. Niemeijer, O. Nishikawa, D. Prior, K. Sauer, M. Savage, A. Schleicher, D. R. Schmitt, N. Shigematsu, S. Taylor-Offord, D. Teagle, H. Tobin, R. Valdez, K. Weaver, T. Wiersberg, J. Williams, N. Woodman, M. Zimmer, Extreme hydrothermal conditions at an active plate-bounding fault. *Nature*. **546**, 137–140 (2017).

46. J. Li, Q. Zhang, T. Yu, M. Zhang, J. Zhang, L. Qiao, T. Wang, R-DTS with Heat Transfer Functional Model for Perceiving the Surrounding Temperature. *IEEE Sens. J.* **20**, 816–822 (2020).
